# Simple New Method for the Preparation of La(IO_3_)_3_ Nanoparticles

**DOI:** 10.3390/nano10122400

**Published:** 2020-11-30

**Authors:** Zoulikha Hebboul, Amira Ghozlane, Robin Turnbull, Ali Benghia, Sara Allaoui, Akun Liang, Daniel Errandonea, Amina Touhami, Abdellah Rahmani, Ibn Khaldoun Lefkaier

**Affiliations:** 1 Laboratoire Physico-Chimie des Matériaux (LPCM), Université Amar Telidji de Laghouat, BP 37G, Laghouat 03000, Algeria; z.hebboul@lagh-univ.dz; 2 Department of Material Sciences, Université Amar Telidji de Laghouat, BP 37G, Laghouat 03000, Algeria; amighozchimie8@gmail.com (A.G.); allaouisarasara@gmail.com (S.A.); aminatouhami2016@gmail.com (A.T.); 3 Departamento de Física Aplicada-ICMUV-MALTA Consolider Team, Universitat de València, c/Dr. Moliner 50, 46100 Burjassot (Valencia), Spain; Akun2.Liang@uv.es (A.L.); daniel.errandonea@uv.es (D.E.); 4 Laboratoire de Physique des Matériaux, Université Amar Telidji de Laghouat, BP 37G, Laghouat 03000, Algeria; benghia11@gmail.com (A.B.); ik.lefkaier@lagh-univ.dz (I.K.L.); 5 Laboratoire des Matériaux et Structure des Systèmes Electromécaniques et leur Fiabilité, LMSSEF Université Larbi Ben M’hidi, Oum El Bouaghi 04000, Algeria; rahmaniabdallah02@gmail.com

**Keywords:** nanoparticles, La(IO_3_)_3_, XRD, FTIR, SEM, non-linear optics

## Abstract

We present a cost- and time-efficient method for the controlled preparation of single phase La(IO_3_)_3_ nanoparticles via a simple soft-chemical route, which takes a matter of hours, thereby providing an alternative to the common hydrothermal method, which takes days. Nanoparticles of pure α-La(IO_3_)_3_ and pure δ-La(IO_3_)_3_ were synthesised via the new method depending on the source of iodate ions, thereby demonstrating the versatility of the synthesis route. The crystal structure, nanoparticle size-dispersal, and chemical composition were characterised via angle- and energy-dispersive powder X-ray diffraction, scanning electron microscopy, and Fourier-transform infrared spectroscopy.

## 1. Introduction

Nanoparticles increasingly play a major role in technologies for cancer prevention, diagnosis, imaging, and treatment [1] due to due to their small size facilitating molecular scale interactions, and the fact that their properties can be enhanced via surface conjugation of a variety of chemicals or molecules. For example, multifunctional nanoparticles can contain specific targeting agents in order to optimise imaging applications, in [2] magnetic properties [3], cell-penetrating characteristics, and many other features. Importantly, the design and synthesis of nanoparticles generates the possibility of developing tumour-specific delivery of imaging probes and therapeutic agents [4]. Additional nanoparticle applications include non-linear optics (NLO), which focuses on non-centrosymmetric nanoparticles, which exhibit second-harmonic generation (SHG) and optical properties aside from classical luminescence [5,6]. 

Many different families of materials have been studied in attempt to enhance medical and NLO applications [7,8,9]. Amongst these, metal iodates [10,11,12,13] are highlighted as some of the most promising, not only as NLO materials but also as dielectric materials and because of their unusual bonding properties related to the presence of an electron lone pair on the iodine atom [14,15,16,17,18,19,20,21,22]. Most metal iodates can be obtained via dissolution, recrystallization, or solvothermal syntheses routes. However, one major obstacle in metal iodate synthesis is that they exhibit rich polymorphism, and these synthesis routes do not necessarily produce single phase products. The synthesis route reported in this article reliably produces single phase nanocrystalline samples. 

One metal iodate of particular interest is anhydrous lanthanum iodate La(IO_3_)_3_ [5]. La(IO_3_)_3_ exhibits four polymorphs: α, β, γ, and δ-La(IO_3_)_3_. Of these four polymorphs, α-La(IO_3_)_3_ is known to be non-centrosymmetric and to be the most efficient SHG material [5]. The α-La(IO_3_)_3_ crystal structure was solved by Ok and Halasyaman [21], and it is described by a monoclinic lattice with a unit cell of the *Cc* space group. Structural information and synthesis conditions regarding α-La(IO_3_)_3_ and its other polymorphs [22,23] are summarised in Table 1. In most works in the literature the La(IO_3_)_3_ synthesis was conducted by reacting iodic acid (HIO_3_) and lanthanum chloride (LaCl_3_). α-La(IO_3_)_3_ nanocrystals were previously synthesized using a microwave-assisted hydrothermal method [5], wherein it was found that whilst the concentration of iodic acid can be used to control the particle size, it exacerbates the problem of polymorphism.

## 2. Materials and Methods

In this work, we present a simple new chemical route to prepare nanoparticles of pure α-La(IO_3_)_3_ and pure δ-La(IO_3_)_3_. In contrast with the hydrothermal method, which combines La_2_O_3_ or LaCl_3_∙6H_2_O with iodic acid (HIO_3_), we have used a soft-chemistry method, which utilises the reaction of sodium iodate (NaIO_3_) and lanthanum nitrate (La(NO_3_)_3_). To the best of the authors knowledge, this method has never been used before. The sample morphology, crystal structure, and optical properties are characterised via: angle- and energy-dispersive powder X-ray diffraction, scanning electron microscopy, and Fourier-transform infrared spectroscopy. The influence of substituting NaIO_3_ with HIO_3_ as the iodate ion source will also be discussed.

The traditional synthesis of the anhydrous metal iodates La(IO_3_)_3_ and Cd(IO_3_)_2_ in aqueous or nitric acid solutions uses a chloride salt source of metal ions (LaCl_3_) added to an acidic iodate source (HIO_3_) according to reaction (1):

LaCl_3_ + 3HIO_3_ → La(IO_3_)_3 (crystalline)_ + 3HCl. (1)

Reaction (1) reveals the remarkable structural polymorphism of La(IO_3_)_3_ and Cd(IO_3_)_2_, which exhibit with four and six polymorphs, respectively [22,24,25]. However, reaction (1) does not produce single-phase samples, likely due to the presence of HCl as a by-product, which is not ideal for structure determination or selective chemistry applications. The presence of [Cl]^−^ in these mixtures can trigger an unwanted oxidation–reduction reaction: 2HIO_3_ + 10HCl ⇌ I_2_ + 6H_2_O + 5Cl_2_.

The new synthesis route reported in this work, reaction (2), avoids the problematic polymorphism by first synthesising an X-ray amorphous sample, which is subsequently heat treated. The choice of reactants also avoids the HCl by-product, and neither HIO_3_ nor NO_3_^−^ cause problematic oxidation–reduction reactions. The synthesis uses a soft-chemistry method, in which a nitrate salt source of lanthanum (La(NO_3_)_3_) is added to an iodate source (NaIO_3_ or HIO_3_):

La(NO_3_)_3_ + 3NaIO_3_ (3HIO_3_) → La(IO_3_)_3 (X-ray amorphous)_ + 3NaNO_3_ (3HNO_3_) (2)

Moderate heat treatment of the X-ray amorphous La(IO_3_)_3_ samples at 400 °C for two hours produces single-phase samples of the α-La(IO_3_)_3_ (δ-La(IO_3_)_3_) polymorph when the acidic source of iodate is NaIO_3_ (HIO_3_). Sample preparation methods for α- and δ-La(IO_3_)_3_ are summarised in Table 2. 

α-La(IO_3_)_3_ nanoparticles were prepared by precipitation of lanthanum nitrate hexahydrate (La(NO_3_)_3_∙6H_2_O, Sigma Aldrich, 98%, St. Louis, MO, USA) with sodium iodate (NaIO_3_, Sigma Aldrich, 99%, St. Louis, MO, USA), which were used without further purification. The reagents were separately dissolved in water at room temperature respecting a 3:1 molar ratio ([IO_3_]^−^:[La]^+^). Sodium iodate was first dissolved into water (0.59 g in 10 mL H_2_O), which was then added to the lanthanum nitrate solution (0.43 g in 2 mL of H_2_O). The reaction of the mixture was spontaneous, precipitating a white powder of La(IO_3_)_3_, which, according to XRD, was amorphous (Product 1A, yield 84.5%). After filtration and washing with deionized water, Product 1A was finally heat-treated at 400 °C in tubular furnace for two hours giving nanocrystals of α-La(IO_3_)_3_ (Product 2A). It is important to highlight that with our method α-La(IO_3_)_3_ can be synthesized in hours, rather than days as is the case with the hydrothermal method.

δ-La(IO_3_)_3_ nanoparticles were prepared following a very similar route using dissolved iodic acid (HIO_3_, Sigma Aldrich, St. Louis, MO, USA, 99.5% purity, 0.53 g in 6 mL H_2_O) instead of sodium iodate. The precipitation reaction was not spontaneous in this case. After three days under slow evaporation at 60 °C, a white powder of La(IO_3_)_3_ was precipitated (Product 1B, yield 56.6%). XRD also indicated that this powder was amorphous. After filtration and washing with deionized water, Product 1B was finally heat-treated at 400 °C in a tubular furnace for two hours producing nanocrystals of δ-La(IO_3_)_3_ (Product 2B).

Phase purity was assessed in ambient conditions via X-ray powder diffraction (XRD) performed on a Philips X’pert Pro Advance diffractometer (Almelo, Netherlands), Cu K_α1_ radiation λ = 1.54056 Å, 40 mA, 40 kV) in the 10–120° range for α-La(IO_3_)_3_ and in the 20–60° range for δ-La(IO_3_)_3_. A step size of 0.01° was used with an acquisition time of 6 s/step.

The nanoparticle size-dispersion and chemical composition were checked by scanning electron microscopy (SEM) using a TESCAN VEGA3 SBU EasyProbe electron microscope system (Brno, Czech Republic) attached with a Bruker detector (Billerica, MA, USA) for energy dispersive X-ray (EDX) analysis. The molar contents of lanthanum and iodine were determined using the ESPRIT Microanalysis Software from Bruker. Secondary electron images were recorded using 5 and 8 keV primary electrons. 

The interaction of infrared (IR) radiation with La(IO_3_)_3_ was studied by means of Fourier-transform infrared (FTIR) spectroscopy using a FTIR Jasco FT/IR-4200 instrument (Tokyo, Japan) with a resolution of 4 cm^−1^. The FTIR spectrum was recorded with a range of 4000–500 cm^−1^ in the transmission configuration using a KBr pellet as the sample carrier.

## 3. Results

### 3.1. Morphology and Composition

**Figure 1:**
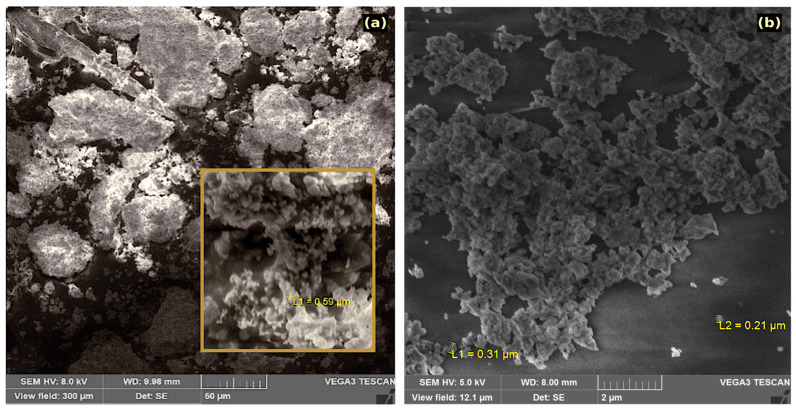
SEM images of agglomerations of La(IO_3_)_3_ nanocrystals. (**a**) α-La(IO_3_)_3_ nanocrystals. The inset shows the simple with higher magnification. (**b**) δ-La(IO_3_)_3_ nanocrystals.

Figure 1 shows SEM images of α- and δ-La(IO_3_)_3_ (products 2B and 2B). The resolution of the SEM instrument did not allow for ideal imaging at the nanometre scale; however, it did allow for the acquisition of images of nanoparticle agglomerates. It was also possible to characterise the chemical composition and the homogeneity of the synthesized powders. The micrograph in Figure 1a shows that the α-La(IO_3_)_3_ sample consisted of micron sized spherical agglomerations of nanoparticles, the diameter of agglomerations smaller than 590 nm in diameter. One such agglomeration is highlighted in the inset of Figure 1b. Energy-dispersive X-ray spec0troscopy (EDX) was used to confirm the composition and phase purity of the prepared α-La(IO_3_)_3_. Within the limits of experimental error, EDX analyses by both weight percent and atomic percent of lanthanum and iodine were found to be in agreement with their corresponding expected molar ratio of 1:3. Figure 1b shows a micrograph of the δ-La(IO_3_)_3_ sample. The estimated diameter of the smaller δ-La(IO_3_)_3_ agglomerations is between 210 and 310 nm, labelled L1 and L2 in Figure 1b. In this case, EDX also confirmed a 1:3 molar ratio between lanthanum and iodine. The presence of impurities was not detectable either α-La(IO_3_)_3_ or δ-La(IO_3_)_3_.

### 3.2. Powder X-ray Diffraction

**Figure 2:**
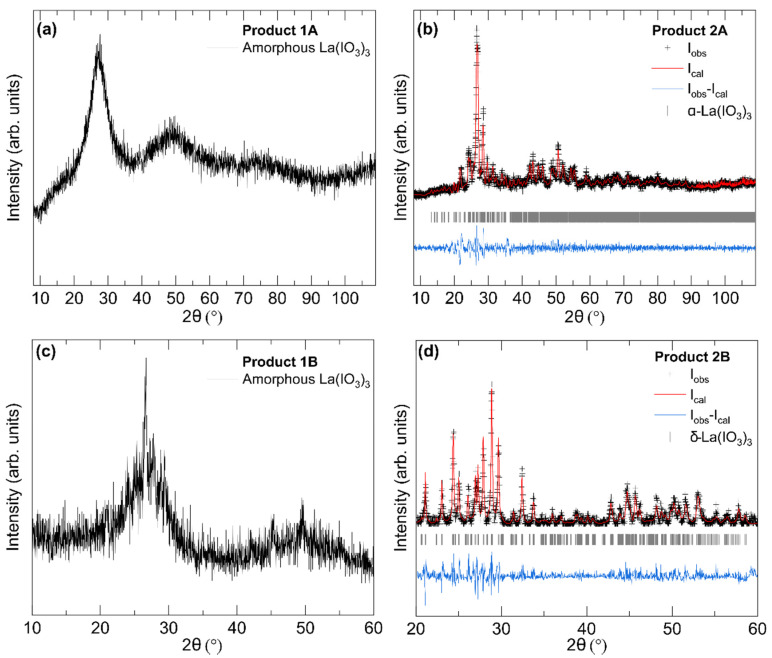
Integrated X-ray diffraction patterns of amorphous and crystalline La(IO_3_)_3_. (**a**) Product 1A, (**b**) α-La(IO_3_)_3_ (Product 2A), (**c**) Product 1B, and (**d**) δ-La(IO_3_)_3_ (Product 2B). For the crystalline phases (**b**,**d**), the experimental data are shown with black crosses, the refinements with red lines, and the residuals with blue lines. Ticks show the calculated positions of Bragg peaks.

Figure 2 displays the results of XRD measurements on the precursor amorphous samples (Products 1A and 1B) and nanocrystalline α-La(IO_3_)_3_ and δ-La(IO_3_)_3_ samples obtained after the thermal treatment (Products 2A and 2B). As displayed in Figure 2a, the integrated XRD pattern from Product 1A exhibits very broad reflections, indicating that the material is X-ray amorphous with only short-range ordering. Product 1B (Figure 2c) exhibits very similar broad reflections with a few additional sharp peaks around 27, 45, and 50°, indicating that the sample is largely amorphous but with a more semi-crystalline nature than Product 1A. XRD patterns acquired after the thermal treatment show clear sample recrystallization. The XRD patterns of Products 2A and 2B (shown in Figure 2b,d) exhibit only sharp reflections, indicating the purely crystalline character of the obtained nanocrystals. The different reflections observed in the XRD patterns shown in of Figure 2b,d clearly distinguish the two different crystal structures of α-La(IO_3_)_3_ and δ-La(IO_3_)_3_.

The broad diffuse humps in the X-ray patterns of amorphous phases (Figure 2a,c) correspond to the distribution of interatomic distances in the short-range structural order of the amorphous [26]. In Product 1A, the most intense reflection is found to be at 3.23 Å. The next most intense reflection is found to be at 1.87 Å. These two distances are, respectively, and roughly correspond to typical La-O and I-O bond lengths in α-La(IO_3_)_3_, so they probably are related to the first-coordination sphere of La and I atoms. In the case of Product 1B the broad reflections correspond to distances of 3.29 and 1.82 Å, respectively.

Products 2A and 2B were identified as α-La(IO_3_)_3_ and δ-La(IO_3_)_3_ by means of a Rietveld and Le Bail refinement, respectively. The refinements are displayed in Figure 2b,d. The obtained unit-cell parameters are summarized in Table 1, and they are in good agreement with those reported in the literature [21,22]. In the case of α-La(IO_3_)_3_ (Product 2A), the XRD pattern can be assigned to the monoclinic non-centrosymmetric space group *Cc*. This was verified by means of a Rietveld refinement, in which we used the atomic positions of Ref. [21] and only refined the unit-cell parameters. The refined converged to small R-factors: *R_p_* = 3.27% and *R_wp_* = 4.92%. This, and the small residuals of the refinements (see Figure 2b), indicates a correct structure identification. No secondary phase or impurities can be detected from the XRD or aforementioned EDX measurements.

In the case of Product 2B, all peaks can be indexed using the orthorhombic centrosymmetric space group (*Pmmm*) proposed by Taouti et al. [22]. Unfortunately, this structure has not been solved yet, and consequently, the atomic positions remain unknown. Therefore, a Rietveld refinement could not be performed; however, a LeBail fit (Figure 2d) leads to small residuals and R-values: *R_p_* = 6.32% and *R_wp_* = 8.13%. The obtained unit-cell parameters (Table 1) agree with the literature [22] and Product 2B can confidently be assigned to the δ-polymorph of La(IO_3_)_3_.

Notice that the α-polymorph of La(IO_3_)_3_ was obtained when NaIO_3_ was used as source of the iodate ion, and the δ-polymorph of La(IO_3_)_3_ was obtained when HIO_3_ was the source of the iodate ion. Therefore, the choice of the acid used to trigger the formation of La(IO_3_)_3_ is crucial for obtaining the desired polymorph.

Finally, by using the Scherrer formula applied to the Full-width at half-maxima (FWHM) of the main Bragg peaks of α-La(IO_3_)_3_ the mean particle size was estimated to be 58(5) nm. In the case of δ-La(IO_3_)_3_ the mean particle size was estimated to be 45(5) nm. 

### 3.3. Fourier-Transform Infrared Spectroscopy

**Figure 3:**
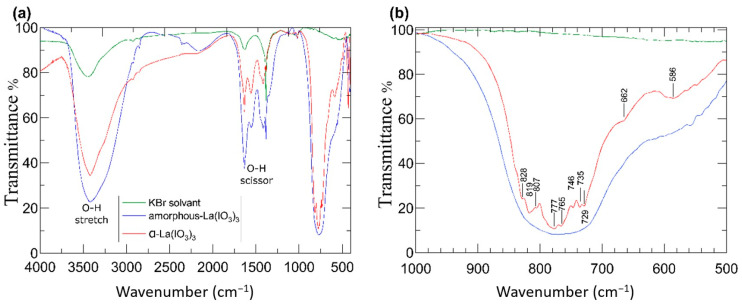
FTIR transmission spectra of amorphous-La(IO_3_)_3_ (blue), α-La(IO_3_)_3_ nanocrystals (red), and KBr (green). (**a**) In the 4000–500 cm^−1^ range and (**b**) in the 1000–500 cm^−1^ range. Ticks and corresponding labels indicate position of the absorption peaks discussed in the text.

The sample transparency and response to infrared (IR) excitation are very important for NLO applications, particularly those which take advantage of SHG such as laser applications. Since α-La(IO_3_)_3_ is an SHG material and δ-La(IO_3_)_3_ is not, the IR properties of α-La(IO_3_)_3_ only have been characterised here. The white colour of the sample is consistent with a band gap in the ultraviolet [27] in particular with the 3.2 eV value reported in the literature [21]. The IR transmission spectrum of α-La(IO_3_)_3_ in the 4000–500 cm^−1^ region is displayed in Figure 3a. Figure 3b shows the 1000–500 cm^−1^ region with greater resolution to facilitate the identification of modes associated with the iodate anion. In the FTIR spectrum, there are contributions from H_2_O absorption bands around 1280–1650 and 3400 cm^−1^ [20]. The most relevant information for α-La(IO_3_)_3_ is in the 1000–500 cm^−1^ region, which is not affected by H_2_O absorption bands.

**Figure 4:**
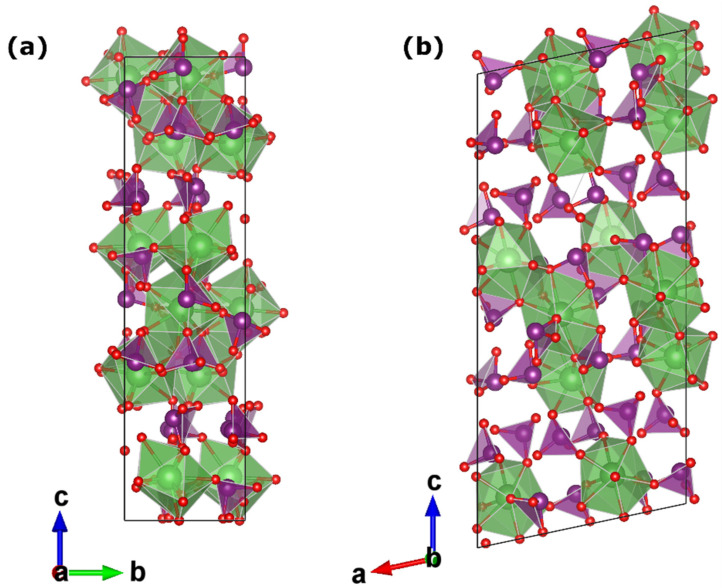
The crystal structure of α-La(IO_3_)_3_. (**a**) Projection along the *a*-axis. (**b**) Projection along the *b*-axis. Lanthanum coordination polyhedra are shown in green. The IO_3_ polyhedra, which exhibit a trigonal-pyramidal configuration, are shown in purple. Oxygen atoms are shown in red.

The infrared absorption spectra were measured in the amorphous La(IO_3_)_3_ sample (Product 1A, blue spectrum) and the crystalline α-La(IO_3_)_3_ sample (Product 2A, red spectrum). The spectra qualitatively share most of the absorption features; however, the crystalline α-La(IO_3_)_3_ structure obtained after the heat treatment exhibits distinguishable absorptions bands in the 1000–500 cm^−1^ region where amorphous La(IO_3_)_3_ only exhibits a broad absorption band. The absorption features in α-La(IO_3_)_3_ are at 586, 662, 729, 733, 746, 777, 807, 819, and 828 cm^−1^, and they are denoted by ticks in Figure 3a. The frequencies of these absorptions agree well with previous results from single crystals samples of α-La(IO_3_)_3_ [28,29,30]. The observed absorptions are typical of iodates [31] and can be correlated with internal vibrations of the IO_3_ polyhedra, which are nearly isolated in the crystal structure of α-La(IO_3_)_3_. To better illustrate to the reader, the crystal structure of α-La(IO_3_)_3_ is shown in Figure 4. The structure has 12 formula unit per unit-cell, and 234 vibrational modes are expected for this structure according to group theory (117A′ + 117A′′). Of the total 234 modes, two A′ modes and one A′′ mode are the acoustic modes, and all of the optical modes both Raman and IR active, which makes mode assignment not a trivial task. However, the fact that the crystal structure of α-La(IO_3_)_3_ consists of large LaO9 polyhedra connected by isolated asymmetric IO3 polyhedra could help with the discussion of vibrational modes. Notice that the iodine atoms are linked to three oxygen atoms in a distorted trigonal-pyramidal environment (see Figure 4). The I-O bonds are short in comparison with the La-O bonds, exhibiting an average value of 1.796 Å, while the average La-O distance is 2.620 Å. Consequently, the vibrational spectra of α-La(IO_3_)_3_ can be interpreted in terms of high-frequency internal modes associated with the IO_3_ polyhedron, in which its centre of mass does not move, and lower frequency modes involving movements between IO_3_ as a rigid unit and La atoms. By analogy with other iodates and oxides [20,31,32,33], the modes at 828–729 cm^−1^ can be assigned to symmetric and asymmetric stretching modes of the IO_3_ polyhedron. This is also consistent with the fact that the modes at 828–729 cm^−1^ correspond to the strongest absorptions of α-La(IO_3_)_3_. Additionally, the absorption bands at 662 and 586 cm^−1^ could be ascribed to bending modes of IO_3_, with some lattice mode contributions involving vibrations between La and IO_3_ as a rigid unit. 

## 4. Discussion

We report here a new and cost-effective synthesis route towards α-La(IO_3_)_3_ nanocrystals, which are predicted to be high performance IR NLO nanoparticles with optical biomarker applications. The method uses a soft chemistry technique, which produces the desired nanoparticles in a matter of hours, compared to the more common hydrothermal technique, which requires days. The synthesis of α-La(IO_3_)_3_ nanoparticles, which exhibit NLO properties, is the first to use NaIO_3_ as an iodate source. The use of HIO_3_ as an iodate source favours the formation of δ-La(IO_3_)_3_, thus demonstrating the versatility of the synthesis technique, which can target a single polymorph product.

The synthesized samples have been characterized by X-ray diffraction, scanning electron microscopy, EDX, and FTIR spectroscopy. SEM analysis facilitated identification of nanocrystal agglomerations. The crystal structure and chemical composition of nanocrystals were confirmed by XRD and EDX. Additionally, XRD enabled the determination of average nanocrystal to be 58 and 43 nm for α-La(IO_3_)_3_ and δ-La(IO_3_)_3_, respectively. Finally, FTIR spectroscopy allowed the determination of IR-active phonons in α-La(IO_3_)_3_, which has been discussed.

## Figures and Tables

**Table 1:** Summary of synthesis conditions, space-group, and lattice parameters of the α-, β-, γ-, and δ-polymorphs of La(IO_3_)_3_. Nanocrystal samples are indicated.

Polymorph	Synthesis Conditions	SG	Unit-Cell Parameters	Ref.
α-La(IO_3_)_3_	Hydrothermal treatment, 220 °C (4 days): La_2_O_3_ + 14 HIO_3_ in water	*Cc*	*a* = 12.526(2) Å, *b* = 7.0939(9) Å, *c* = 27.823(4) Å, *β* = 101.975(4)°	[21]
	Hydrothermal treatment, 220 °C (4 days): LaCl_3_∙6H_2_O + 4 HIO_3_ in water		*a* = 12.4920 Å, *b* = 7.0720 Å, *c* = 27.7270 Å, *β* = 102°	[22]
	Nanocrystals Microwave-assisted hydrothermal method, 250 °C (1 h) LaCl_3_ + 3 HIO_3_ in water		*a* = 12.5454 Å, *b* = 7.0939 Å, *c* = 27.8304 Å, *β* = 102.044°	[5]
	Nanocrystals Present work		*a* = 12.57(1) Å, *b* = 7.102(7) Å, *c* = 27.69(3) Å, *β* = 101.7(1)°	This work
β-La(IO_3_)_3_	Thermal decomposition at 490 °C: La(IO_3_)_3_-(HIO_3_) or La(IO_3_)_3_-(HIO_3_)_1.33_	*P*2_1_	*a* = 7.2539(4) Å, *b* = 8.5360(5) Å, *c* = 13.5018(7) Å, *β* = 97.499(2)°	[22]
γ-La(IO_3_)_3_	Reversible transition from β-La(IO_3_)_3_ at 140 °C. Cannot be recovered at room temperature	*P*2_1_/*c*	*a* = 7.3427(9) Å, *b* = 8.684(1) Å, *c* = 13.741(2) Å, *β* = 99.913(8)°	[22]
δ-La(IO_3_)_3_	Thermal decomposition at 300 °C: La(IO_3_)_3_-(HIO_3_) Thermal decomposition at 340 °C La(IO_3_)_3_-(HIO_3_)_1.33_	*Pmmm*	*a* = 10.3646(6) Å, *b* = 10.3758(6) Å, *c* =15.4933(6) Å	[22]
	Nanocrystals Present work		*a* = 10.35(1) Å, *b* = 10.36(1) Å, *c* =15.45(2) Å	This work

**Table 2:** Summary of chemical preparation and heat-treatment conditions for α- and δ-La(IO_3_)_3_. The yield of Products 2A and 2B is the same as Products 1A and 1B, respectively, because there is no loss of sample through heat treatment.

Reaction	Reagents	Temperature, Time of Reaction	Yield %	Product 1	Heat Treatment Temp, Time	Product 2
**A**	La(NO_3_)_3_∙6H_2_O + 3NaIO_3_ (0.43g) (0.59g)	Room temperature, spontaneous	84.5	amorphous-La(IO_3_)_3_	400 °C, 2 h	Nanopowder α-La(IO_3_)_3_
**B**	La(NO_3_)_3_∙6H_2_O + 3HIO_3_ (0.43g) (0.53g)	60 °C, 3 days	56.6	amorphous-La(IO_3_)_3_	400 °C, 2 h	Nanopowder δ-La(IO_3_)_3_
